# Increasingly separate spheres? The changing state gender discourse in China from 1990 to 2023

**DOI:** 10.3389/fsoc.2026.1777050

**Published:** 2026-05-29

**Authors:** Jia Wang, Ruiyu Zhang

**Affiliations:** 1 Department of Applied Social Sciences, The Hong Kong Polytechnic University, Hung Hom, Hong Kong SAR, China; 2 Department of Politics and Public Administration, University of Hong Kong, Pokfulam, Hong Kong SAR, China

**Keywords:** China, family, gender roles, inequality, state, work

## Abstract

This study examines how state gender discourse in China has evolved over the past three decades, with a focus on how official narratives construct women’s roles in the public and private spheres. Although research has documented a resurgence of traditional gender ideology and worsening gender inequality in post-reform China, less is known about how state-controlled media have framed womanhood over time. Using computational text analysis on 125,532 People’s Daily news articles published between 1990 and 2023, we trace shifts in the semantic associations between women and public- versus domestic-role vocabularies. Our analysis shows that, beginning in the late 1990s and intensifying through the 2010s, official discourse increasingly distanced women from public identities while maintaining a strong and relatively stable emphasis on their domestic roles. This growing asymmetry reflects a discursive move toward “increasingly separate spheres,” in which women’s identities are more tightly anchored in the family than in public life. These findings clarify how central state rhetoric has adapted to market reforms and welfare restructuring, and highlight the role of official discourse in articulating gender boundaries and reproducing gendered expectations in contemporary China.

## Introduction

After the founding of the People’s Republic of China, the state adopted a strongly “anti-traditional” stance and promoted absolute equality between men and women, actively encouraging women’s participation in all spheres of public life (Zhang et al., 2025). Recent research indicates that gender discourse in Chinese media has shifted toward more traditional norms during the marketization era (Sun and Chen, 2015).^1^ Large-scale surveys show that Chinese people increasingly endorse traditional views about gender roles (Xu, 2016), especially within the family (Jia and Ma, 2015; Qian and Li, 2020). This resurgence of traditional gender ideology has coincided with persistent—and in some cases widening—gender gaps in both the workplace and household labor (Wu, 2019). Studies document growing inequality in earnings, occupational segregation, discrimination, and promotion opportunities (He and Wu, 2017, 2018; Wang and Wong, 2021; Yang and Xie, 2021; Zhang et al., 2008). At home, women continue to perform the majority of housework and childcare (Ji et al., 2017; Wu, 2019). The latest Global Gender Gap Report ranks China 106th out of 146 countries, underscoring a troubling decline in gender equality since the onset of market reform (World Economic Forum, 2024).

Scholars attribute this revitalization of traditional gender ideology and the rise in gender inequality to a combination of factors, including economic marketization (He and Wu, 2017; Wang and Wong, 2021), the growing culture of consumerism and the commodification of women (Yang, 1999), and the retreat of the state from social provisioning (Gu, 2013; Wu, 2009, 2010). While economic and cultural explanations have received considerable attention, the role of the state remains equally critical. As market reforms have advanced, the Chinese central government has reoriented its gender discourse from state-led socialist equality toward a market-centered “quality (*sushi*)” framework, weakening the institutional support for women’s rights and interests (Wu, 2010).^2^ In China’s top-down governance structure, the way the central government frames womanhood in both domestic and public spheres plays a pivotal role in shaping public gender discourse and, consequently, the broader landscape of gender inequality.

Despite the recognized significance of state-level gender rhetoric, prior research has not systematically examined how state-controlled media have framed women’s roles in the public versus private spheres over time. This is a significant gap, given the central government’s capacity to shape public opinion and societal attitudes toward gender through its official discourse (Acker, 1992; Dangoisse and Perdomo, 2021; Dobrowolsky and Jenson, 2004). In China, state-controlled media and propaganda apparatuses profoundly influence national conversations about gender, social norms, workplace expectations, and family responsibilities. Analyzing content published by the state’s flagship press therefore offers a crucial lens for understanding the mechanisms behind the resurgence of traditional gender ideology, the persistence of gender inequalities, and the ways these themes are communicated and reinforced in contemporary Chinese society. This study addresses this gap by systematically tracing the evolution of state gender discourse and providing a detailed account of how official narratives have framed women’s roles in the public and private spheres over the past three decades.

Building on Ji et al. (2017), this study adopts a “two-sphere separation” framework to analyze the evolving boundaries between the public and private spheres in Chinese state gender discourse. Ji et al. (2017) use this concept refers to describe a historically specific, state-driven process in which the previously integrated public and private spheres under socialism become institutionally and ideologically decoupled: social production is increasingly governed by the market, while social reproduction is re-privatized within families, especially onto women.^3^ This framework highlights how the retreat of the state from socialist welfare and egalitarian ideology has enabled a resurgence of traditional gender norms, with the state now actively endorsing the re-privatization of care and the centrality of women’s domestic roles. In particular, disadvantages faced by women in the labor market and within the family are mutually reinforcing, a dynamic that is both reflected in and perpetuated by state discourse. In this configuration, the shift from Marxist egalitarianism toward a hybrid of revived Confucian patriarchy and neoliberal market logic recasts women’s unpaid care work and domestic responsibilities as natural and desirable, even as women remain formally encouraged to participate in paid employment and public life.

By systematically quantifying the positioning of women within the state agenda—whether emphasizing their roles in the home or celebrating their professional achievements—this study brings empirical evidence to bear on theoretical claims about two-sphere separation and ideological transformation in the government’s gender discourse over time. Drawing on 125,532 articles published between 1990 and 2023 in *People’s Daily*, the official newspaper of the Central Committee of the Chinese Communist Party (CCP), we employ computational text analysis to track shifts in the semantic relationships of words associated with women in the public and private spheres. By documenting these discursive shifts, our research contributes to understanding how state rhetoric is reconfiguring gender norms and how these changes are embedded in the broader context of gender inequality in contemporary China.

## The grand transition: from “private embedded in the public sphere” to “increasingly separate spheres”

The pre-reform era in China was characterized by a strongly “anti-traditional” stance on gender roles, often referred to as “state feminism” (Wang, 2005; Wu, 2009, 2010; Zhang et al., 2025). Following the founding of the People’s Republic of China, official gender rhetoric—rooted in Marxist feminist principles—actively championed women’s participation across all areas of public life, epitomized by the slogan “*Women hold up half the sky*” (Tong, 2022). During the planned economy period, the state played a proactive role in advancing gender equality in employment and in the broader social division of labor, advocating absolute equality between men and women in both legislation and ideology (Zhang et al., 2025). For example, the iconic image of the “*iron girls*” in the 1960s and 1970s exemplifies the government’s radical efforts to “degender” various sectors of public labor (Jin, 2006).

Although the socialist state recognized household labor as an important form of social labor that supported production outside the home (Song, 2011), state-led emancipation primarily targeted women’s participation in the public sphere and only partially transformed gender relations within the family. Despite significant gains in women’s public participation, the division of household labor remained largely traditional, with most women bearing a heavy “second-shift” burden of housework, childcare, and eldercare (Ji et al., 2017; Zuo and Bian, 2001). Overall, the socialist era was characterized by a pattern of “private embedded in the public sphere” (Song, 2011, 2012), in which women’s domestic roles were taken for granted even as their public roles were celebrated.

At the same time, Maoist gender ideology had uneven reach and impact across social and spatial contexts. State feminist projects were most intensively implemented in urban work units, state-owned enterprises, and some collective production brigades, where political campaigns, workplace regulations, and propaganda more directly pressed women into waged labor and public life (Wang, 2005; Yang, 1999). In many rural and peripheral areas, however, the penetration of this rhetoric was more limited, and local cadres often compromised between egalitarian ideals and entrenched patrilineal, patrilocal family structures. Even where the state strongly promoted women’s public participation, it invested far less effort in redistributing care or transforming masculinity; women’s “double burden” of full-time work and primary responsibility for housework and care remained largely unchallenged in both policy and everyday practice (Ji et al., 2017; Liu, 2007; Zuo and Bian, 2001). In this sense, pre-reform “state feminism” was oriented more toward mobilizing women for production than restructuring domestic gender relations, leaving substantial ideological and practical space for traditional expectations of women’s familial responsibilities to persist beneath a socialist egalitarian veneer.

Ji et al. (2017) argues that post-reform gender inequality must be understood against this backdrop. As Marxist egalitarian ideology waned, the previously integrated arrangement gave way to what they term “two-sphere separation”: social production became increasingly organized by market institutions, while social reproduction and care were shifted back to families and, in practice, to women. This institutional and ideological decoupling of the public and private spheres generates a configuration in which women’s disadvantages in the labor market and within the family mutually reinforce one another.

Since the onset of the market reform in 1978, the state’s discourse on gender roles has undergone gradual and complex transformations (Hare, 2016; Ji and Wu, 2018). As market forces reconfigured labor opportunities and media narratives, the Chinese government retreated from its earlier commitment to absolute gender equality and comprehensive social provisions for women, allowing more traditional gender ideas and practices to resurface (Cao and Hu, 2007; Gu, 2013; Liu, 2007). Rather than merely acquiescing to market and cultural forces, the state has actively orchestrated the separation of public and private spheres, endorsing traditional gender norms through policy and propaganda (Ji et al., 2017). This separation is not simply a descriptive contrast between “work” and “family,” but a state-led restructuring of institutions and meanings that reassigns care to the private sphere and redefines appropriate femininity, a process that should be observable in the content and emphases of state gender discourse over time.

These discursive transformations occurred alongside major changes in women’s socioeconomic position and in the organization of care. On the one hand, women’s educational attainment increased substantially during the post-reform period. Women benefited relatively more from higher education expansion (Wu and Zhang, 2010), and the proportion of women obtaining tertiary education (two-year college and above) doubled from 26.2% in 1985 to 53.4% in 2021 (Ministry of Education of the People’s Republic of China, 2021). Chinese women also continued to participate in paid employment at comparatively high levels, despite a slight decline in labor force participation since 1990 (Hare, 2016; International Labour Organization, 2021). On the other hand, these gains did not translate into full equality in the public sphere. Gender gaps in earnings, occupational attainment, promotion opportunities, and employment security persisted and, in some domains, widened with marketization. Meanwhile, women continued to shoulder the majority of unpaid domestic labor and care work within families (Wu, 2019).

These patterns were reinforced by the restructuring of socialist welfare institutions, particularly in urban China. Under the planned economy, work units and collective institutions provided some childcare, medical care, pensions, and other social services, partially socializing the costs of reproduction. With market reform, many of these supports were weakened, privatized, or shifted back to households. As a result, care responsibilities were increasingly re-familialized, intensifying women’s double burden of paid work and unpaid domestic labor (Ji et al., 2017). This institutional context is crucial for understanding why state discourse about women’s public and private roles matters: official rhetoric did not emerge in isolation, but alongside structural changes that maintained women’s labor force participation while relocating social reproduction back into the family.

Against this backdrop, we anticipate a two-sided transformation in the Chinese state’s gender discourse during the post-reform period. First, China’s transition to a market-oriented economy fundamentally reshaped the public realm. The rise of the private sector and the increasing dominance of market principles—such as efficiency and productivity—transformed the labor market, often to the detriment of women’s participation and advancement. As economic reforms deepened, the state gradually retreated from its previous role as a direct provider of social protection and services, including childcare, medical care, and pensions. This withdrawal shifted greater responsibility for social reproduction onto individuals and families, thereby altering the context in which gender roles are constructed and negotiated. In this context, it is reasonable to expect that state rhetoric would gradually move away from promoting women’s active participation in public life: the Chinese state would become increasingly less likely to frame womanhood in terms of women’s public roles during the post-reform period.

Second, in the aftermath of economic reforms, the private sphere also underwent significant transformation, with the reproduction functions—once partially socialized—falling back to the family (Sun and Chen, 2015). This re-privatization of care and domestic labor has been accompanied by several notable waves of public debate over “women returning home,” each reflecting societal anxieties and shifting expectations about women’s social roles (Song, 2011). These debates have periodically reignited discussions about whether women should prioritize family responsibilities over participation in the workforce.

Since the 2010s, the Chinese state has moved beyond passive acquiescence to these trends and has actively begun to endorse an ideology that reemphasizes women’s domestic responsibilities and the centrality of the family (Song and Gao, 2022). This ideological shift is evident in official rhetoric and policy initiatives that promote traditional family values and encourage women to embrace domestic roles. For example, despite its longstanding commitment to gender equity in education, employment, health, and other public arenas, the Chinese government has recently stressed women’s roles in promoting family education and values in official propaganda. In *The Outline for the Development of Chinese Women (2021–2030)*, one of the primary goals is to “encourage and support women to play their unique roles in family life; … to lead their family members in actively participating in activities for building a civilized family, a ‘Five-Good’ family, the most beautiful family, and other mass spiritual civilization construction activities” (State Council of the People’s Republic of China, 2021). Although the outline formally supports gender equality in household work, its asymmetrical emphasis on women’s role in shaping family values—without corresponding mention of men’s responsibilities—may nonetheless reinforce traditional gender attitudes that constrain women within the family. Based on these developments, we expect a broader ideological shift toward traditional gender norms, with the Chinese state increasingly framing womanhood in terms of women’s domestic roles in the post-reform period, especially in recent years.

**Figure 1:**
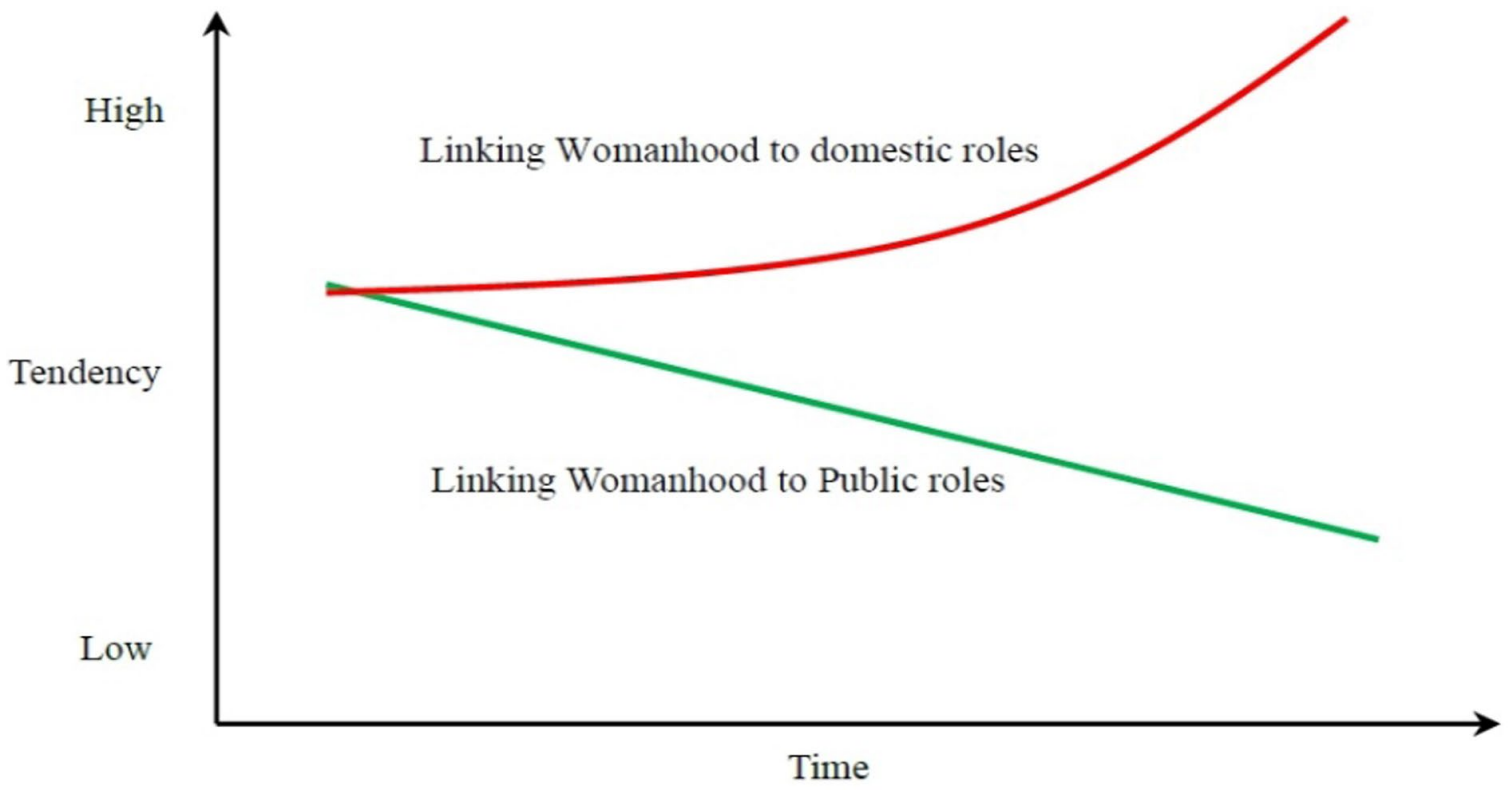
The hypothesized two-sided shift in the Chinese state gender discourse during the post-reform period.

Figure 1 summarizes this hypothesized two-sided shift in Chinese state gender discourse during the post-reform period. The vertical axis represents the tendency to link womanhood to particular roles (from low to high), and the horizontal axis represents time. The red line indicates a growing likelihood of associating womanhood with domestic roles, suggesting that, over time, the state is expected to emphasize women’s domestic responsibilities more strongly. In contrast, the green line depicts a declining tendency to link womanhood to public roles, indicating a decreasing emphasis on women’s participation in public life. Together, these trends depict a post-reform era of “increasingly separate spheres,” marked by stronger state endorsement of traditional, family-centered gender norms.

## Methods

### Data and sample

Our data are drawn from all published news articles in *People’s Daily* between 1990 and 2023 (totaling 1,183,285 articles). As the official newspaper of the CCP, *People’s Daily* directly reflects the viewpoints of the CCP and the central government on a range of gender issues in China. While the newspaper’s large corpus spans a period of significant economic and sociopolitical transformation, coverage from the early 1990s onward is particularly valuable for examining how government discourse on women has evolved in response to market reforms, family policies, and shifts in media strategy.

To identify articles addressing women and their roles in both public and private spheres, we constructed several lists of keywords in three steps. First, drawing on existing scholarship on women’s work, family, and social reproduction in China, along with our own close reading of *People’s Daily* articles from different periods, we compiled an initial pool of candidate terms. Second, we examined high-frequency words and collocates in a stratified sample of articles that mentioned neutral references to women across decades to identify recurrent terms associated with women’s public and domestic roles. Third, we iteratively refined the lists (e.g., removing ambiguous items, consolidating clear synonyms, and reassigning terms whose predominant usage did not match the intended category) through repeated inspection of sample articles and consultation with experts on Chinese media discourse and gender politics. Specifically, we first developed three groups of keywords: (1) neutral terms referring to women without connotations of domestic or professional status (11 terms); (2) “public sphere” terms encompassing common words associated with women’s roles, abilities, and performance in the workplace (17 words); and (3) “private sphere” terms describing women’s roles related to marriage, caregiving, or household responsibilities (17 words) (Appendix Table S1). We then screened for articles containing any of these broadly defined keywords, resulting in a dataset of 125,532 articles (totaling 103.2 million tokens) from 1990 to 2023.

For robustness checks, we also constructed two alternative groups of 15 keywords representing women’s traditional temperament and another 15 keywords denoting modern temperament following the same steps (see Appendix Table S2), and used these to screen and analyze additional articles. This yielded a supplementary dataset of 62,446 articles (totaling 65.1 million tokens). This approach builds on existing literature that distinguishes between evolving ideals of femininity in China—ranging from traits such as independence, ambition, and assertiveness, which are often associated with modern professional identities, to more traditional values like gentleness, obedience, and nurturing, historically linked to domestic and family roles (Sun and Chen, 2015).

### Measuring gender discourse using word embeddings

We employ a meta-embedding text analysis approach that combines Word2Vec with Continuous-Bag-of-Words (Word2Vec-CBOW) and FastText models to analyze semantic trends in our selected corpus of news articles. Word embeddings, which are learned from word co-occurrences in large text corpora, represent the meanings of words as coordinate vectors in a continuous, high-dimensional meaning space—where proximity between vectors indicates similarity (Boutyline and Arseniev-Koehler, 2025). Notably, word embeddings trained on popular media corpora can serve as computational models of the cultural schemas inherent in those texts (Boutyline et al., 2023).

While the standard Word2Vec model has proven effective in studies of English and other alphabetic languages, Chinese text presents unique challenges due to its logographic writing system and morphological complexity. FastText, developed to improve upon the skip-gram model (Bojanowski et al., 2017), represents each word as a collection of character n-grams, making it particularly advantageous for Chinese, where many words are formed by combining characters with distinct meanings. However, FastText’s strength in handling out-of-vocabulary tokens and rare terms may sometimes come at the expense of capturing nuanced contextual relationships, which Word2Vec’s established training protocols (skip-gram or continuous bag-of-words) can better highlight. Given these complementary strengths, we adopt a meta-embedding strategy (Hossain et al., 2023) that combines both techniques by averaging the Word2Vec and FastText embeddings for each token. This approach leverages the advantages of both models, yielding more robust and stable representations for a morphologically complex language like Chinese. In particular, the meta-embedding mitigates cases where a token is well-represented by one model but not the other, or where FastText’s morphological breakdowns require the semantic anchoring that Word2Vec provides. The result is a refined, hybrid vector space in which tokens can be compared and clustered with greater confidence in their contextual relationships.

For preprocessing, we use *jieba*, a widely recognized Chinese text segmentation library, to tokenize the data. The total number of words and articles per year is reported in Appendix Table S3. We then independently train models on each year’s corpus and repeat this process across decades to capture potential shifts in the semantic meanings of women’s roles. This approach ensures that the embeddings reflect yearly variations in vocabulary and discourse—an important consideration in an evolving media landscape where key terms, official slogans, or policy language may rise and fall in prominence. By operating at the yearly level, our analysis captures fine-grained temporal dynamics, reducing the risk that short-lived but meaningful shifts are obscured by a single, monolithic model spanning multiple decades.

In the resulting models, semantically or syntactically similar words are located closer to each other than dissimilar words, as measured by cosine similarity. Words with the highest proximity are those that either frequently co-occur or can be used interchangeably, even if they rarely appear together (e.g., synonyms) (Mrkšić et al., 2016). We use cosine similarities between neutral keywords and the two groups of keywords (private and public spheres) to measure the strength of association in the embeddings. Higher values (closer to 1) suggest that references to women are more closely associated with a particular domain—either the domestic or public sphere—while lower values suggest weaker associations (Garg et al., 2018). For example, a high neutral-private cosine similarity indicates that official rhetoric closely aligns women’s identity with familial roles, whereas a lower value implies a discursive distancing of womanhood from domestic responsibilities. Similarly, the neutral-public similarity indicates the extent to which references to women are linked to the workplace at different points in time.

Across our study period, the average cosine similarity between gender and the private sphere is 0.80 (range: 0.70 to 0.88), while the average similarity between gender and the public sphere is 0.60 (range: 0.39 to 0.85), suggesting a closer association between gender and domestic life than with public life. The standard deviation (SD) of the cosine similarity between gender and the public sphere (0.13) is larger than that for the private sphere (0.05), indicating greater variability in the association between gender and public life compared to private life. We calculate the difference between the gender-private and gender-public cosine similarities as our key measure of the Chinese central government’s tendency to frame womanhood in relation to the domestic versus the public sphere. Higher values indicate a more traditional gender disposition by the state. We refer to this difference as the “Relatively Conservative Gender Rhetoric” (RCGR) exhibited by the Chinese state.

## Results

### Shifts in women’s public and private role associations: descriptive patterns

**Figure 2:**
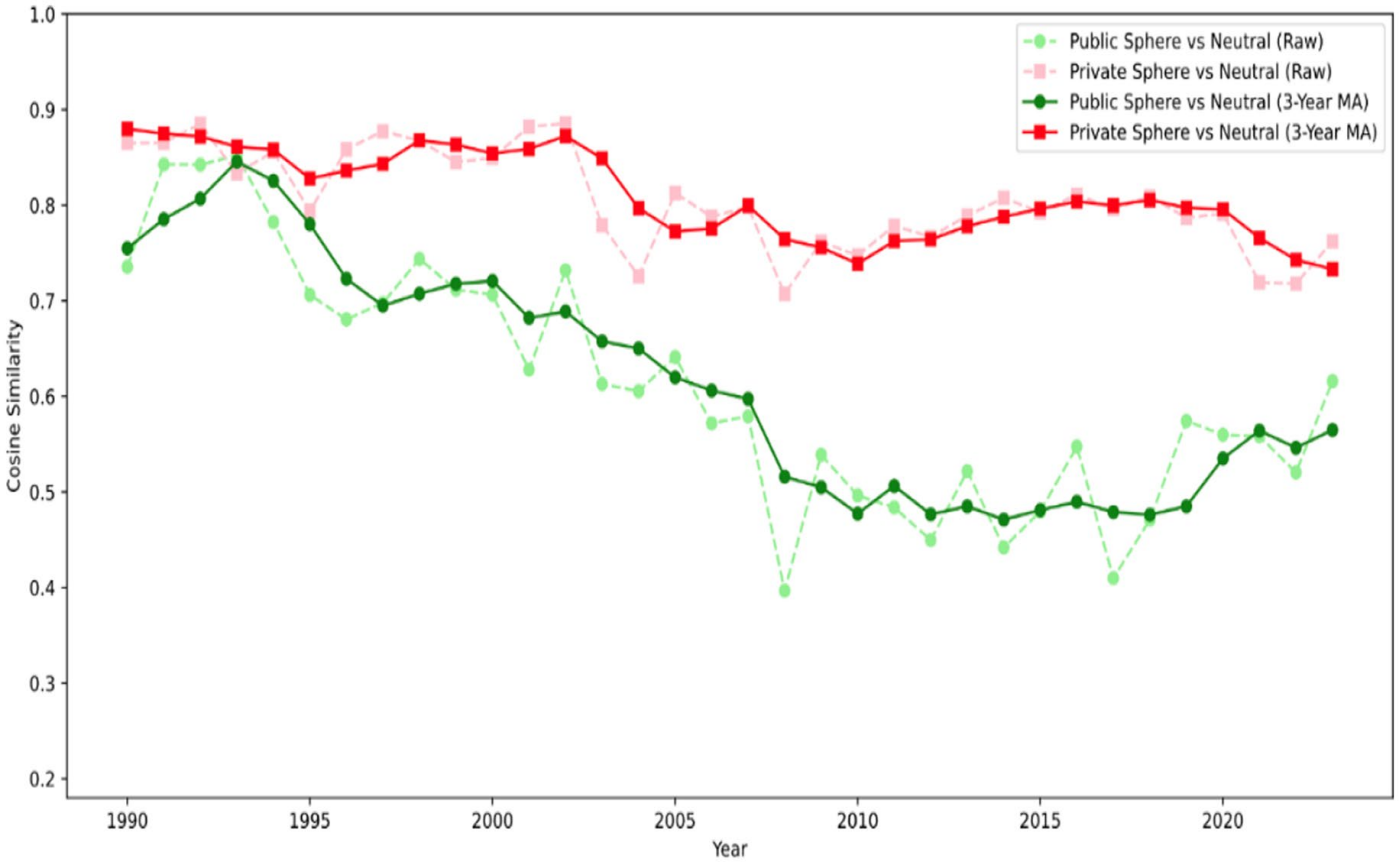
The associations (cosine similarities) between gender and the public versus private spheres, 1990–2023.

Yearly meta-embeddings reveal substantial shifts in the semantic positioning of women-related concepts in *People’s Daily* from 1990 to 2023. As shown in Figure 2, throughout the entire period the semantic center of “women” has been more closely associated with private-sphere themes than with public-sphere themes. Specifically, in the early 1990s, private-neutral similarity scores were consistently high (above 0.83), indicating that government references to women were strongly aligned with familial, caregiving, and domestic roles. Over the same period, public-neutral similarity scores rose steadily, peaking at 0.84 in 1993. At that point, the gap between private- and pubic-neutral similarity narrowed to just 0.02, suggesting that associations between women and public versus private spheres were nearly equivalent. This pattern reflects an intertwined framing of women’s public and private roles in early post-reform state discourse, largely inherited from the socialist era.

From the late 1990s onward, these trends began to diverge. Private-neutral similarity remained relatively stable, with only a slight decline beginning in the early 2000s, whereas public-neutral similarity dropped sharply until around 2010 and then plateaued. Consequently, the gap between private- and public-neutral similarity widened over time. Between 2008 and 2018, public-neutral similarity fell below 0.50, reaching a low of 0.43 in 2012, while private-neutral similarity remained high (above 0.73). This widening gap—peaking at 0.34 in 2018—points to a decisive rhetorical shift, where women were increasingly distanced from the public sphere while remaining firmly anchored in domestic and familial roles.

From 2020 onward, we observe a modest rebound in public-neutral similarity and a narrowing gap (e.g., 0.17 in 2023). This suggests a cautious reintroduction of public-oriented representations of women, potentially in response to changing labor demands or evolving discursive strategies related to gender equality.

Table 1 reports the average values of private-neutral and public-neutral similarities across seven time windows from 1990 to 2023. The association between womanhood and domestic roles (private-neutral similarity) remained largely unchanged before 2005; it declined slightly thereafter, but the magnitude of this change was limited. By 2023, the link between gender and private life remained strong, with a similarity score of 0.752. In contrast, the association between gender and public roles, which was similarly high in the early 1990s (0.806), declined significantly over time, shrinking by 32%. The RCGR, which captures the gap between the two measures, increased steadily, indicating that Chinese state gender discourse has become more conservative and progressively less likely to frame women’s roles in terms of their participation in public life.

**Table 1:** Average associations between private-neutral and public-neutral keywords as well as their changes over time.

Time window	Private-neutral similarity (1)		Public-neutral similarity (2)		Relatively conservative gender rhetoric (3) = (1)–(2)	
	Mean	Change	Mean	Change	Mean	Change
1990–1994	0.868		0.806		0.062	
1995–1999	0.848	−0.02	0.722	−0.084**	0.126	0.064*
2000–2004	0.848	−0.02	0.670	−0.136***	0.179	0.116***
2005–2009	0.776	−0.092***	0.563	−0.243***	0.212	0.150***
2010–2014	0.766	−0.102***	0.479	−0.327***	0.287	0.225***
2015–2019	0.796	−0.072***	0.476	−0.330***	0.320	0.258***
2020–2023	0.752	−0.116***	0.549	−0.256***	0.202	0.140***

*p < 0.05, **p < 0.01, ***p < 0.001 (two-tailed tests). Change is from the average associations during 1990–1994.

These patterns are consistent with a broader trend toward “increasingly separate spheres,” driven primarily by the weakening association between womanhood and public roles. Given the relatively stable—albeit slightly decreasing—association between womanhood and domestic roles, our theoretical expectation of a two-sided shift in Chinese state gender discourse (as depicted in Figure 1) is not supported. Instead, the evidence points to a growing separation produced mainly by a one-sided change in the public sphere.

### Interpreting the patterns: institutional and ideological contexts

Taken together, these findings suggest that the semantic repositioning of women in state discourse is tightly intertwined with broader institutional and ideological shifts. The brief convergence of public- and private-sphere associations in the early 1990s reflects a transitional moment when Mao-era “half the sky” rhetoric still legitimized women’s participation in production, and rapid economic restructuring sustained strong demand for women’s labor. For example, a 1992 article titled “Women should play their ‘half the sky’ role on the main battlefield of economic construction” in *People’s Daily* (1992-03-08, page 3) explicitly calls to “mobilize and organize women of all ethnic groups and all walks of life to participate in economic development… demonstrating the ‘half the sky’ role of Chinese women as key contributors to economic construction.” At the same time, women were praised as holding “the key to family happiness” because of their “special contribution to family life” (“Women’s family status on the same horizon,” *People’s Daily*, 1995-08-20, page 1). During this phase, it remained discursively coherent for official media to celebrate women simultaneously as workers and as family pillars, and our embeddings capture that integrated framing.

The subsequent divergence, driven mainly by a declining association between womanhood and public roles, coincides with the deepening of market reforms and welfare restructuring. As work-unit–based services such as childcare and other social supports were curtailed or marketized, the costs of social reproduction were increasingly shifted back onto households (Ji et al., 2017; Wu, 2010). At the same time, state discourse moved away from a socialist equality frame toward a “quality” (*suzhi*)-oriented logic that cast women as responsible for raising high-quality children and maintaining family stability (Gu, 2013; Sun and Chen, 2015). Coverage in the mid-2000s to 2010s foregrounds women’s double burden and intensifying care pressures. One article notes that the 330 million women “working diligently in various jobs across the country make up half of China’s total workforce” and “shoulder the dual responsibilities of family and career” (“Children have the right to drink their mother’s breast milk,” *People’s Daily*, 2004-03-23, page 10).^4^ Another piece on second births reports that “a mother’s energy and the need for dedicated care for children before they attend kindergarten are key factors affecting the decision to have a second child… She resigned and stayed at home when her child entered the first grade of primary school” (“*Giving birth is easy, raising children is difficult*,” *People’s Daily*, 2017-01-20, page 19). In this context, maintaining a strong discursive link between women and domestic roles while gradually weakening the link to public roles is consistent with a broader project of re-familializing care and responsibilizing women for social reproduction under market conditions.

The partial rebound of public-role associations after 2020 emerges against a different backdrop. China now faces pronounced demographic challenges, such as low fertility, rapid population aging, alongside persistent labor market pressures and rising precarity (Wang and Gerber, 2023). Recent policy documents and propaganda materials increasingly call for the “comprehensive development” of women, highlight women’s “indispensable role” in sectors such as public health, disaster relief, and innovation, and promise expanded childcare to help women “balance” family and career (State Council of the People’s Republic of China, 2019, 2021). For instance, a 2020 article states that “whether in critical and urgent moments such as epidemic prevention and control, flood fighting and disaster relief, or in daily work and life including economic development, political construction, culture and education, women from all walks of life play an indispensable and vital role” (“*Comprehensive development, holding up half the sky*,” *People’s Daily*, 2020-09-15, page 6). A 2022 piece calls for “attach[ing] greater importance to the development of female human capital… develop[ing] inclusive childcare services to reduce families’ parenting burdens… [and] enabl[ing] women to better balance family and career” (“*Give better play to the role of ‘half the sky’ in the new era and new journey*,” *People’s Daily*, 2022-03-25, page 5). The modest uptick in public-sphere associations observed in our embeddings aligns with this recalibration: the state does not abandon a familized vision of womanhood, but supplements it with renewed appeals to women’s economic and civic contributions.

### Robustness check

**Figure 3:**
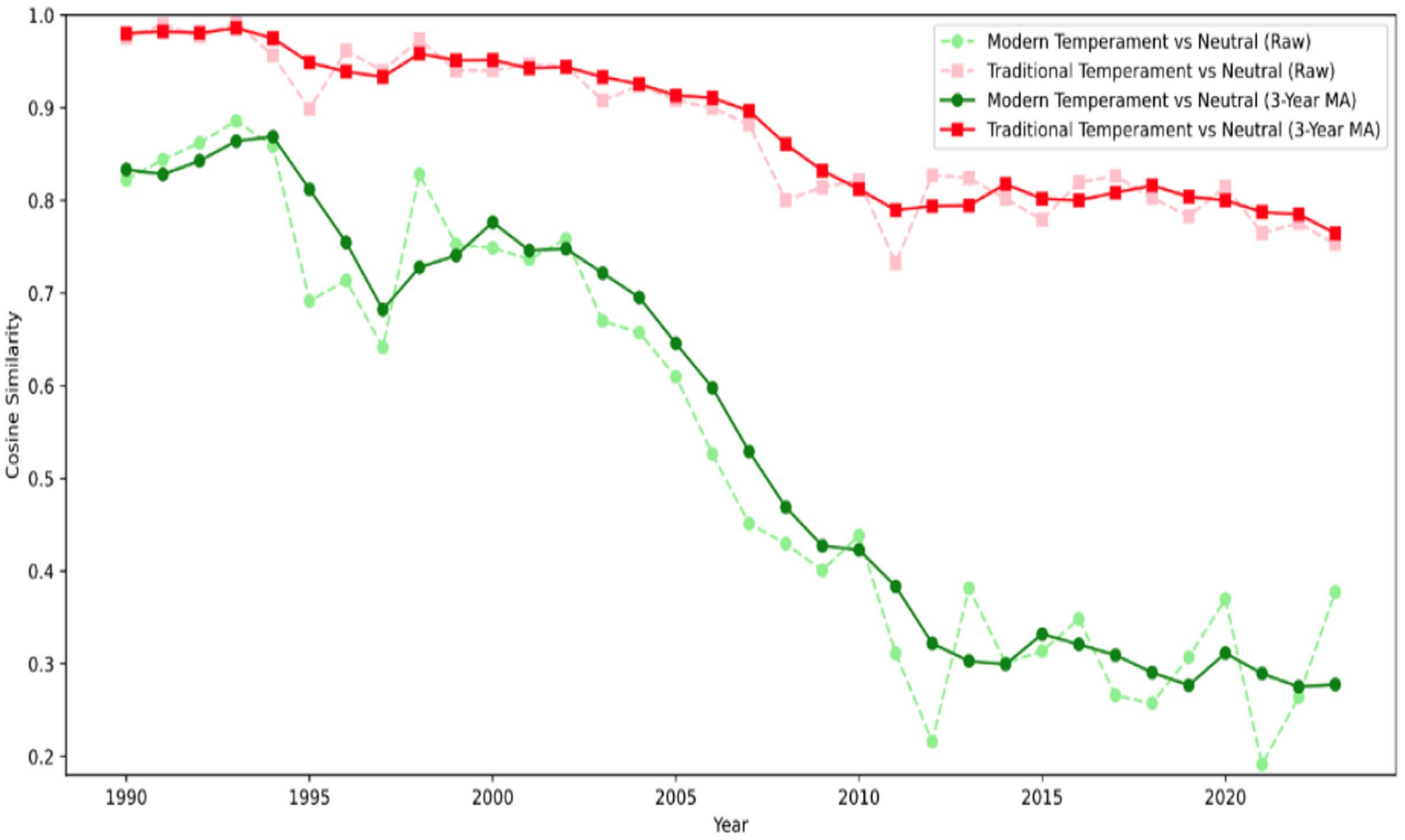
The associations (cosine similarities) between gender and modern versus traditional temperament, 1990–2023.

To assess the robustness of our main findings, we conducted a supplementary analysis using an alternative set of gender-related keywords capturing feminine traits, categorized as either modern or traditional temperament. As shown in Figure 3, cosine similarities between traditional temperament terms and the neutral centroid declined only slightly over time. By contrast, cosine similarities between modern temperament terms and neutral terms remained high from 1990 until the early 2000s, indicating that qualities such as confidence, capability, and assertiveness were closely associated with the generic notion of womanhood in state discourse. Around the mid-2000s, however, there was a noticeable decline in the semantic association between modern traits and the neutral centroid, although this downward trend slowed after 2015.

Consistent with the main analyses, this robustness check again illustrates a broad pattern of “increasingly separate spheres,” driven by a rhetorical retreat from portraying women as assertive or professionalized—traits associated with public roles—in state-sponsored mainstream media during the post-reform period.

## Discussion

This study advances understanding of gender and family in contemporary China by applying and extending the “two-sphere separation” framework (Ji et al., 2017) to three decades of state-controlled gender discourse. Using computational text analysis of *People’s Daily*, we traced how official narratives constructed women’s domestic and public roles across a period of profound social and economic changes. Our analyses yield three key findings.

First, across the entire period, women are consistently more closely associated with the private than with the public sphere. Even as socialist ideology once promoted women’s participation in public life, official discourse has persistently foregrounded women as caregivers and moral anchors within the family. This aligns with scholarship highlighting the enduring cultural resilience of traditional gender norms, even during the Maoist era when policies promoted women’s employment and public participation (Liu, 2007). Although the Maoist slogan “*Women hold up half the sky*” symbolized gender equality, women’s dual burden—public employment combined with domestic duties—remained largely unquestioned (Ji and Wu, 2018). Our empirical evidence confirms that, at the start of the reform period, official rhetoric continued to affirm rather than unsettle traditional familial roles.

Second, we identify a brief period in the early 1990s when references to women are almost equally linked to public and domestic roles. This “dual-role” configuration reflects a transitional moment in which Mao-era gender rhetoric still legitimized women’s participation in production, while market reforms and rapid economic growth sustained strong demand for women’s labor. During this phase, it remained discursively coherent to portray women as both workers and family contributors. This period coincided with accelerated urbanization and the rise of female entrepreneurship, necessitating an official narrative that encouraged women’s involvement in public roles without severing them from traditional familial expectations (Liu, 2007).

Third, from the late 1990s through the 2010s, we observed a growing rhetorical separation: the state increasingly distanced women from public identities while maintaining a strong emphasis on their association with domestic life. This shift signifies a pronounced “reprivatization” of women’s roles in official narratives, resonating with research documenting the resurgence of patriarchal frameworks that positioned women primarily as caregivers and moral custodians rather than equal economic agents (Lazar and Sun, 2020; Sun and Chen, 2015). Our findings also support the argument that the state strategically deploys gender discourse to address social and policy challenges (Ji et al., 2017; Sun and Chen, 2015). After 2020, we observe a modest rebound in public-role associations, suggesting a partial recalibration rather than a return to the earlier integrated “half the sky” discourse. This likely reflects state responses to demographic pressures, labor market needs, and international attention to gender equality. Nonetheless, the overarching pattern is one in which women remain persistently aligned with domestic responsibilities in official discourse.

These temporal patterns map closely onto major institutional and ideological transformations. The early 1990s convergence of public and private associations corresponds to a period when socialist equality rhetoric and work-unit–based welfare still underpinned women’s public participation. As marketization deepened and enterprise-based welfare and childcare services were curtailed or marketized, social reproduction costs were increasingly shifted back to households, and in practice to women (Ji et al., 2017; Wu, 2010). Our results suggest that state discourse adapted to this reconfiguration by preserving a strong semantic link between women and domestic roles while gradually deemphasizing their identities as workers, professionals, or cadres. The sharp divergence between public- and private-sphere associations from the late 1990s through the 2010s is consistent with a broad pattern of “increasingly separate spheres.”

The asymmetric character of this shift is theoretically significant. Much existing work on reprivatization and the resurgence of traditional gender norms in post-reform China emphasizes the revival of domestic ideology (e.g., Wu, 2010; Sun and Chen, 2015). Our analysis refines this picture by showing that the separation between public and private spheres in state discourse occurs primarily through a rhetorical withdrawal from public-role framings rather than a dramatic strengthening of domestic associations. Women’s domestic roles remain strongly emphasized, but not substantially more so than in the early 1990s. Instead, women’s public identities recede from the center of official rhetoric. This pattern helps explain why women’s labor force participation remains relatively high while their public contributions receive limited symbolic recognition: the state continues to rely on women’s work but no longer consistently frames them as equal public subjects.

The modest rebound in public-role associations after 2020 further illustrates the flexibility of state gender projects. Confronted with low fertility, rapid population aging, and persistent labor-market pressures (Wang and Gerber, 2023), recent policy documents and media reports emphasize women’s “indispensable role” in epidemic control, disaster relief, economic development, and governance, while promising expanded childcare to help women “balance” family and career (e.g., State Council of the People’s Republic of China, 2021). Our findings indicate that this is an additive shift: public-role rhetoric is layered on top of an enduring familized ideal of womanhood. The state does not relinquish the expectation that women are primary caregivers; rather, it seeks once again to mobilize them as key contributors to public life, now under a framework that stresses “balancing” rather than equal redistribution of care.

This study is not without limitations. First, our analysis is confined to *People’s Daily*. While this outlet is crucial for understanding state rhetoric, it does not capture the diversity of provincial, commercial, or online media, nor popular reception. Second, the corpus is constructed with theory-driven keyword filters that target texts explicitly marking women and gendered roles. This practice may undercount women’s public roles when they are described in gender-neutral terms and cannot fully distinguish normative endorsements of domesticity from critical or problematizing discussions. Third, word embeddings capture statistical associations between terms, not attitudes or behaviors. Our findings therefore describe shifts in the semantic structure of official discourse rather than direct changes in what people believe or how they act. Finally, our observation window begins in 1990, so we do not capture earlier transitions from Mao-era “state feminism” to the initial reprivatization of family roles. The strong association between womanhood and domesticity in the early 1990s likely reflects ideological shifts that preceded our period of analysis.

Despite these limitations, our study makes several important contributions. Theoretically, we extend and refine the “two-sphere separation” framework of post-reform China (Ji et al., 2017). We demonstrate that separation is not simply a shift toward stronger domestic ideology, but an asymmetric and time-specific process in which the state gradually retracts public-role rhetoric while maintaining high levels of domestic association. We also specify the timing of divergence and partial rebound, linking these phases to marketization, welfare restructuring, and demographic policy. In doing so, we highlight how socialist legacies, Confucian familism, and neoliberal responsibilization are recombined in state discourse to recenter women in the family while selectively invoking their public contributions.

More broadly, our findings underscore that state discourse is an active component of the gender regime. By tracing how official media redefine appropriate femininity as shifting from an integrated public and private ideal to increasingly separate spheres and, more recently, to an emphasis on women “balancing” paid work with family duties, we offer a discursive counterpart to studies of persistent gender gaps in earnings, occupational attainment, and unpaid care. These patterns are consistent with accounts of gender regimes changing in combined and uneven ways (Kandiyoti, 2007, 2014; Kandiyoti et al., 2025; Walby, 2020): elements of a socialist public gender regime, including endorsement of women’s education and employment, coexist with a renewed domesticization of care, morality, and family responsibility. Our semantic measures suggest that the Chinese state continues to support women’s presence in the public sphere, but increasingly reframes it as something to be balanced with intensified household and caregiving obligations, echoing the notion of an incomplete, uneven transition (Walby, 2020) and the idea of a renegotiated patriarchal bargain (Kandiyoti, 2007, 2014; Kandiyoti et al., 2025).

Our findings also speak to feminist political economy research on neoliberal restructuring and social reproduction. Prior studies show that neoliberal reforms in many developing and transitional contexts often reconfigure, rather than simply roll back, state involvement by responsibilizing households—especially women—for managing welfare and risk, sometimes under a rhetoric of opportunity or “empowerment” (e.g., Kunz, 2010). At the same time, such reforms tend to expand women’s role as flexible, low-cost labor while leaving their double burden intact. The Chinese case shares elements of this pattern but, as our study suggests, marketization and responsibilization are articulated with revived Confucian familism and demographic concerns, producing a state discourse that explicitly re-centers women in the family and normalizes the re-privatization of care rather than foregrounding empowerment. In this sense, our results highlight one particular way neoliberal market logic can intersect with local gender ideologies to sustain and deepen women’s double burden.

Methodologically, our study demonstrates how yearly word embeddings, combined with theory-driven keyword sets, can be used to measure long-term changes in state gender discourse in a large Chinese corpus. Our meta-embedding approach, which averages Word2Vec and FastText models, provides a reproducible strategy for tracking evolving semantic associations between women and different social spheres. The robustness check based on modern versus traditional feminine temperament, together with illustrative excerpts from People’s Daily, shows how computational measures can be anchored in qualitative interpretation. This design offers a template for future research on gender, family, and other symbolic boundaries in Chinese and other non-alphabetic languages.

Future research should examine how these rhetorical patterns shape family dynamics, individual aspirations, and policy outcomes, and explore variation across regions, generations, and social classes. By integrating computational methods with relevant social theories, this study illustrates the value of interdisciplinary approaches for analyzing large-scale cultural and ideological change. As China continues to confront demographic and economic challenges, ongoing attention to the evolution of state gender discourse will be essential for understanding the prospects for gender equality and family life.

## Data Availability

The raw data supporting the conclusions of this article will be made available by the authors upon reasonable request.

## Footnotes

1 We discuss the transformation of state gender ideology in more detail in the next section.

2 Many contemporary states adopt pro–women’s rights rhetoric in line with international norms (Htun and Weldon, 2010). In China, however, economic reforms have shifted the internal logic of state gender discourse from a politicized socialist equality project toward a market- and “quality” (*sushi*)-oriented framework. This reorientation has lowered the priority accorded to women’s rights within official discourse, even as the state continues to invoke gender equality in its formal rhetoric (Wu, 2010).

3 In this study, we use “social reproduction” in the sociological and demographic sense to refer to the processes that maintain and renew the population and the labor force on a daily and generational basis, including childbearing, childrearing, household labor, and care for dependents organized through families, workplaces, communities, and the state (Laslett and Brenner, 1989). This usage is compatible with feminist “social reproduction theory,” which conceptualizes social reproduction as structurally intertwined with capitalist accumulation and as a central site of oppression and struggle (e.g., Bhattacharya, 2017; Winders and Smith, 2019). Our primary aim here, however, is not to elaborate a general theory of social reproduction under capitalism, but to trace how the Chinese state discursively organizes the boundary between public production and familial reproduction over time.

4 Excerpts and news articles quoted here, along with additional examples, are summarized in Online Appendix Table S4.
