# Supplementary material for: Increasingly separate spheres? The changing state gender discourse in China from 1990 to 2023
Source: Front Sociol. 2026 May 29;11:1777050. doi: 10.3389/fsoc.2026.1777050 (PMC13259676; doi:10.3389/fsoc.2026.1777050)
Supplement: Supplementary file 1 [file Supplementary_File_1.docx]

**Increasingly Separate Spheres?**

**The Changing State Gender Discourse in China from 1990-2023**

**Online Appendix**

**Table S1** List of keywords.

| Category | Keywords |
| --- | --- |
| Neutral | she, female (three Chinese terms), woman (two Chinese terms), girl, young girl, young woman/lady, sisters, miss |
| Public sphere | female worker, employee, female heroes, Sanba (March 8), half the sky, iron girl, iron lady, women’s army, leading woman, female expert, labor, manual work, job, fighter, model employee/outstanding worker, career, entrepreneurship |
| Private sphere | family, marriage, marry, divorce, childbearing, having children, childrearing, children, offspring, mother and child, family traditions/values, housework, mom, mother, loving mother, virtuous wife, wife |

**Table S2** List of keywords of modern and traditional temperament.

| Category | Keywords |
| --- | --- |
| Neutral | she, female (three Chinese terms), woman (two Chinese terms), girl, young girl, young woman/lady, sisters, miss |
| Modern temperament | agency, self-assuredness, self-mastery, authentic selfhood, self-reliance, equality, optimism, determination, resilience, fortitude, vitality, wisdom, ambition, leadership, professional woman |
| Traditional temperament | gentility, elegance, humility, reserve, virtuosity, submissiveness, compassion, sensitivity, enthusiasm, amiability, tenderness, thoughtfulness, fragility, intellect, dignity |

**Table S3** Number of words and articles per year during 1990–2023.

| **Year** | **Tokens in articles (in 1,000)** | **Articles** |
| --- | --- | --- |
| 1990 | 1930 | 3060 |
| 1991 | 1480 | 2609 |
| 1992 | 1440 | 2574 |
| 1993 | 1380 | 2482 |
| 1994 | 1840 | 2923 |
| 1995 | 2390 | 3823 |
| 1996 | 2230 | 3412 |
| 1997 | 2390 | 3463 |
| 1998 | 2130 | 3342 |
| 1999 | 1980 | 3117 |
| 2000 | 2030 | 3037 |
| 2001 | 1970 | 2952 |
| 2002 | 2000 | 3025 |
| 2003 | 2680 | 4074 |
| 2004 | 2710 | 4119 |
| 2005 | 2720 | 4045 |
| 2006 | 2780 | 3826 |
| 2007 | 2910 | 4032 |
| 2008 | 3210 | 3914 |
| 2009 | 3310 | 3672 |
| 2010 | 4080 | 4672 |
| 2011 | 4880 | 4930 |
| 2012 | 4450 | 4737 |
| 2013 | 4380 | 4921 |
| 2014 | 4320 | 4822 |
| 2015 | 4540 | 4659 |
| 2016 | 4300 | 4376 |
| 2017 | 4450 | 4366 |
| 2018 | 4170 | 4202 |
| 2019 | 3640 | 3470 |
| 2020 | 3820 | 3671 |
| 2021 | 4060 | 3438 |
| 2022 | 3290 | 2892 |
| 2023 | 3310 | 2875 |
| Total | 103200 | 125532 |

**Table S4.** Illustrative *People’s Daily* articles and textual excerpts supporting embedding-based findings.

| **Period and discourse pattern** | **Article title** | **Representative wording** |  |
| --- | --- | --- | --- |
| **Early 1990s** | 在经济建设主战场发挥妇女的“半边天”作用  Women should play their “half the sky” role on the main battlefield of economic construction  Published date: 1992-03-08 (page 3) | “动员和组织各族各界妇女投入经济建设……显示了中国妇女在经济建设主战场的‘半边天’作用。”  "Mobilize and organize women of all ethnic groups and all walks of life to participate in economic development... demonstrating the 'half the sky' role of Chinese women as key contributors to economic construction." | 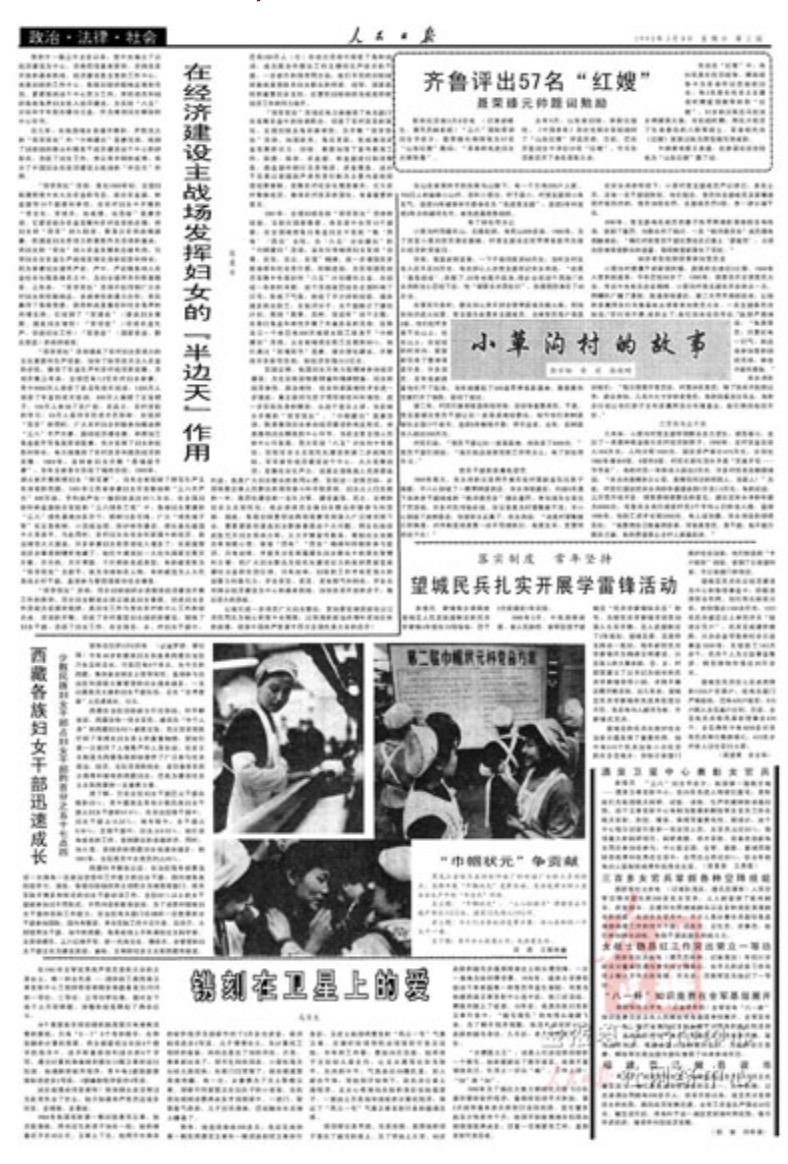 |
|  | 过去女子不如男今朝巾帼“半边天” 妇女投身改革建设展英姿涌现数以百万计的先进模范人物  Women enter reform and construction as “half the sky,” with millions of model figures emerging  Published date: 1992-03-07 (page 1) | “我国5206万女职工积极开展……社会主义劳动竞赛活动，充分显示女职工在社会主义现代化建设和改革开放中的‘半边天’作用。”  "China's 52.06 million female workers actively carried out socialist labor competitions, fully demonstrating the 'half the sky' role of women workers in socialist modernization and reform and opening up." | 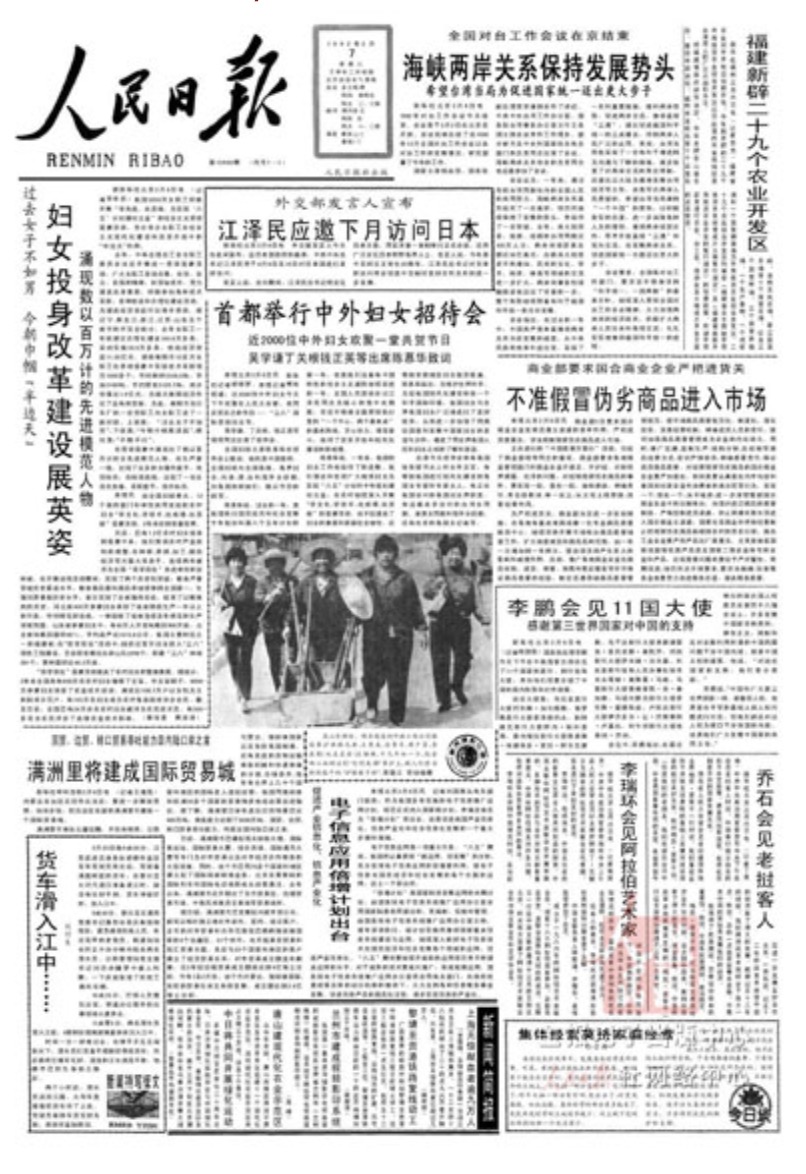 |
|  | 在同一地平线上——中国妇女的家庭地位  Women's family status on the same horizon  Published date: 1995-08-20 (page 1) | “四十五年惊回首，妇女家庭地位的变化地覆天翻……妇女们更由于对家庭的特殊贡献，被社会学家称为掌握家庭幸福钥匙的人。”  "Looking back over forty-five years, women's family status has changed profoundly. Because of their special contribution to family life, women are described as holding the key to family happiness." | 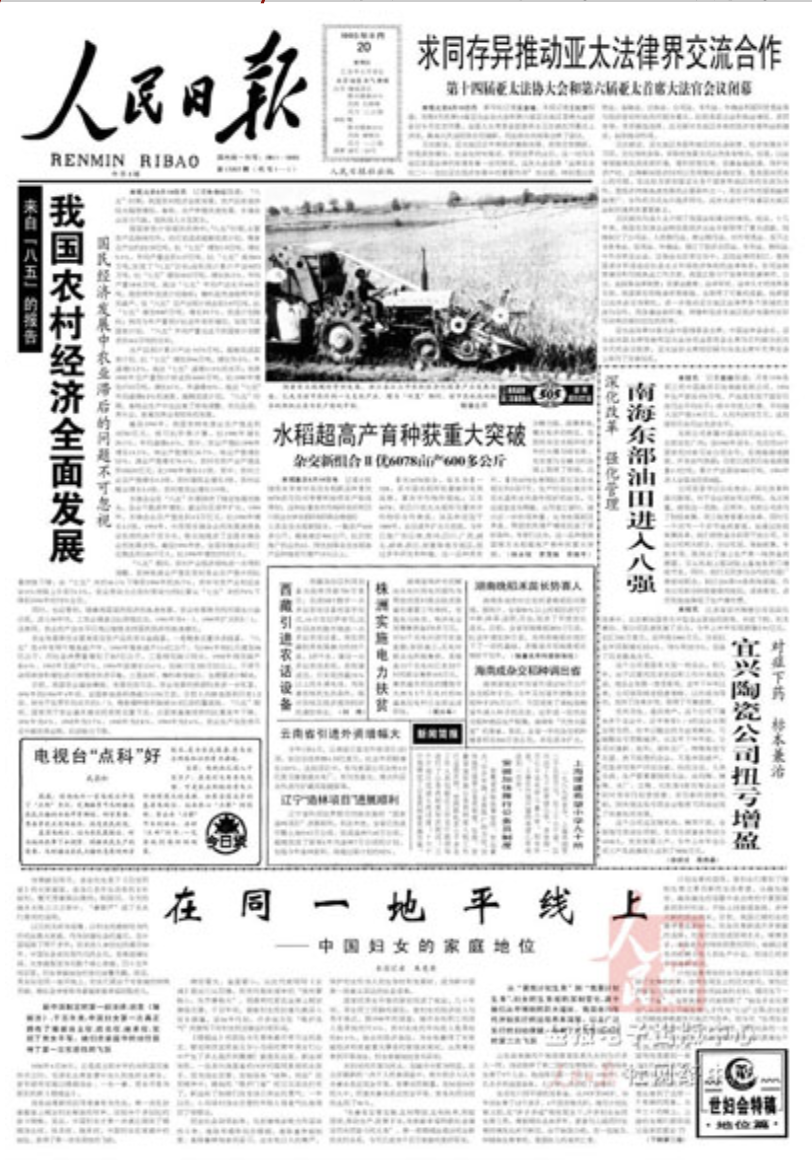 |
|  | 继母情——沈阳军区优秀军人妻子标兵杨秀珍  A stepmother's affection: Yang Xiuzhen, model military wife of the Shenyang Military Region  Published date: 1994-12-16 (page 3) | “有位名叫杨秀珍的母亲——一位军人的遗孀，4个孩子的继母，6位老人的女儿……塑造了一位完美的新时代女性的形象。”  "Yang Xiuzhen was portrayed as a mother, a soldier's widow, a stepmother of four children, and a daughter to six elders, forming the image of a perfect woman of the new era." | 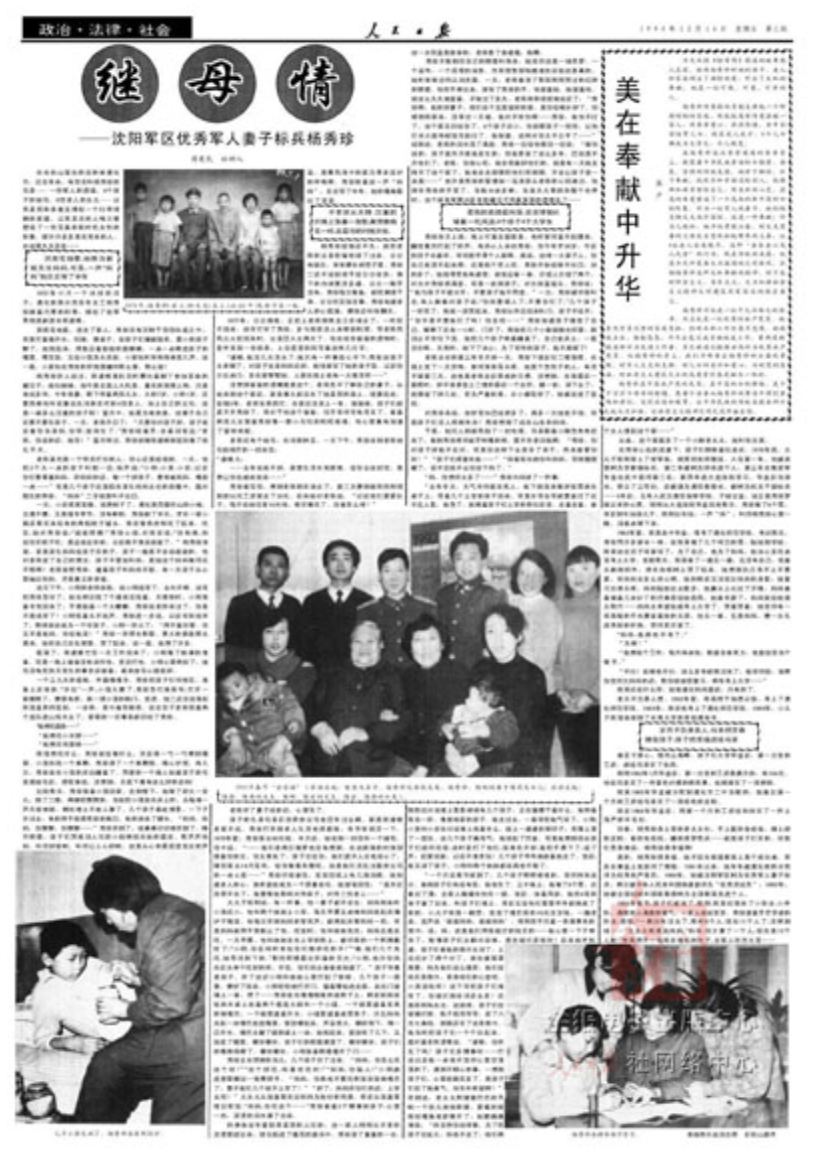 |
| **Late 1990s-2010s** | 孩子有权吮吸母亲的乳汁  Children have the right to drink their mother's breast milk  Published date: 2004-03-23 (page 10) | “我国各种劳动岗位上辛勤工作的3.3亿名妇女，撑起了全国就业人员的‘半边天’。她们……扛起了家庭、工作的双重担子。”  “The 330 million women working diligently in various jobs across the country make up half of China's total workforce. They... shoulder the dual responsibilities of family and career.” | 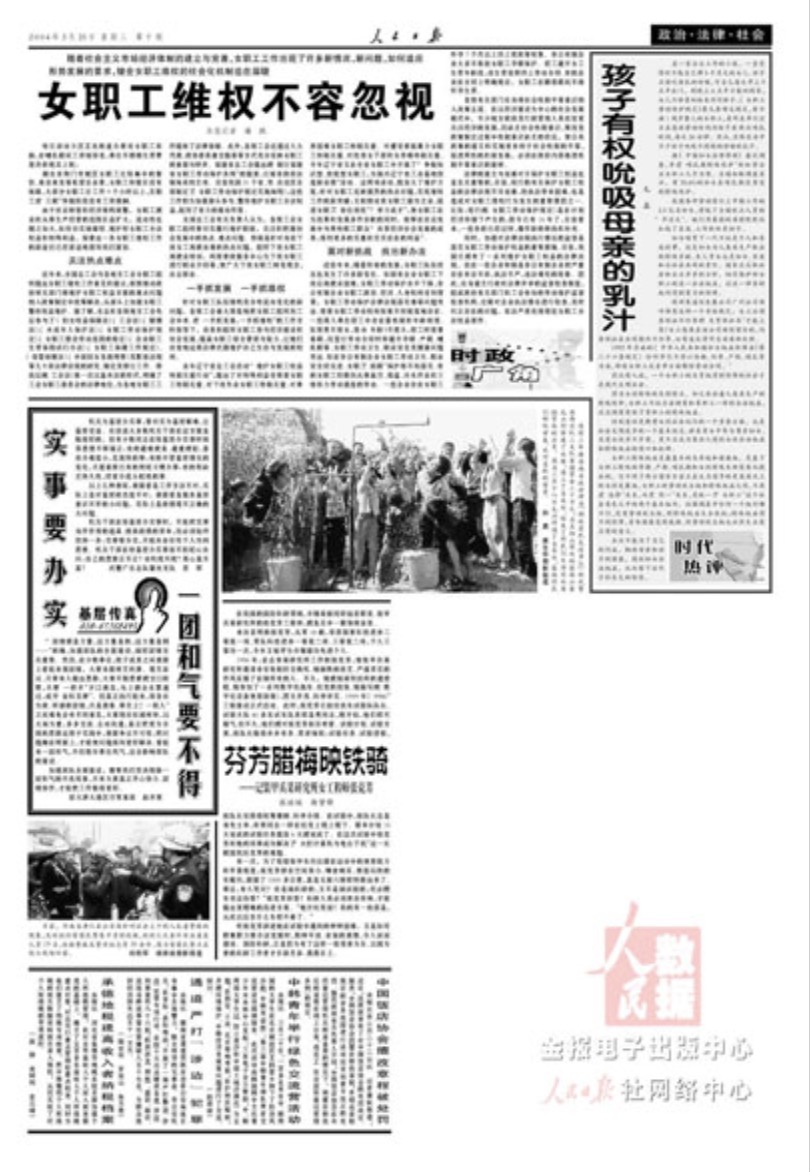 |
|  | 生孩易，养孩难  Giving birth is easy, raising children is difficult  Published date: 2017-01-20 (page 19) | “母亲的精力、孩子上幼儿园以前需专人照料是影响生育二孩的重要因素……孩子上小学一年级时，她辞职回家。”  "A mother's energy and the need for dedicated care for children before they attend kindergarten are key factors affecting the decision to have a second child... She resigned and stayed at home when her child entered the first grade of primary school." | 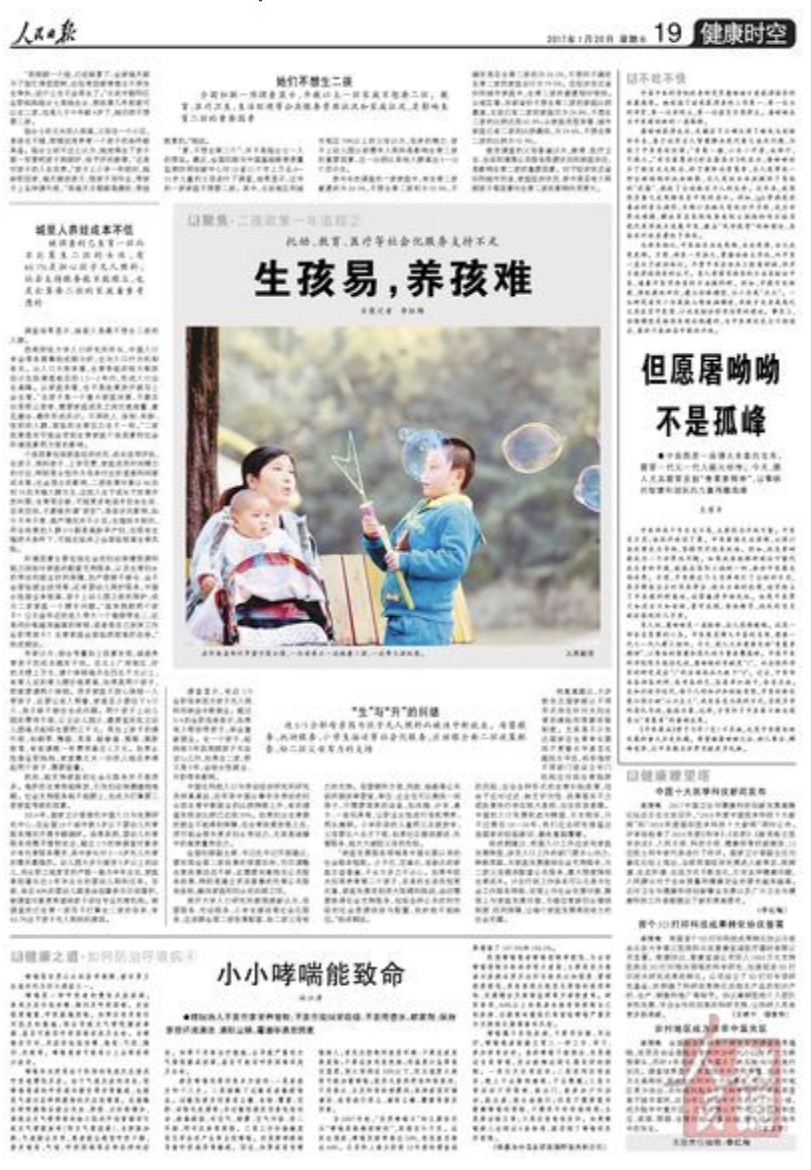 |
|  |  |  |  |
| **Post-2020** | 全面发展 顶起半边天——我国妇女事业成就斐然  Comprehensive development, holding up half the sky: achievements in China's women's cause  Published date: 2020-09-15 (page 6) | “无论是在抗疫、抗洪等急难险重关头，还是经济发展、政治建设、文化教育等日常工作生活中，各行各业女性都发挥着不可或缺的重要作用。”  "Whether in critical and urgent moments such as epidemic prevention and control, flood fighting and disaster relief, or in daily work and life including economic development, political construction, culture and education, women from all walks of life play an indispensable and vital role." | 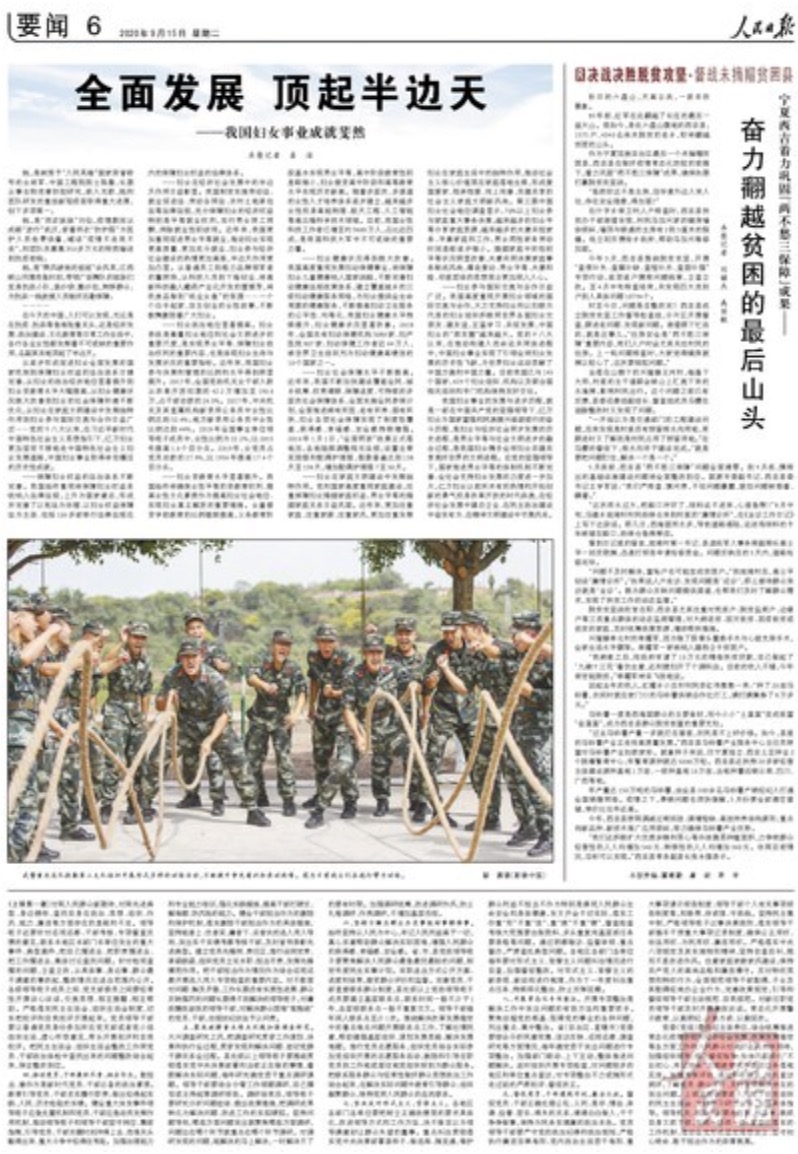 |
|  | 在新时代新征程中更好发挥“半边天”作用  Give Better Play to the Role of "Half the Sky" in the New Era and New Journey  Published date: 2022-03-25 (page 5) | “更加重视开发女性人力资本……发展普惠托育服务，减轻家庭养育负担……让女性能更好平衡家庭和事业。”  "Attach greater importance to the development of female human capital... develop inclusive childcare services to reduce families' parenting burdens... enable women to better balance family and career." | 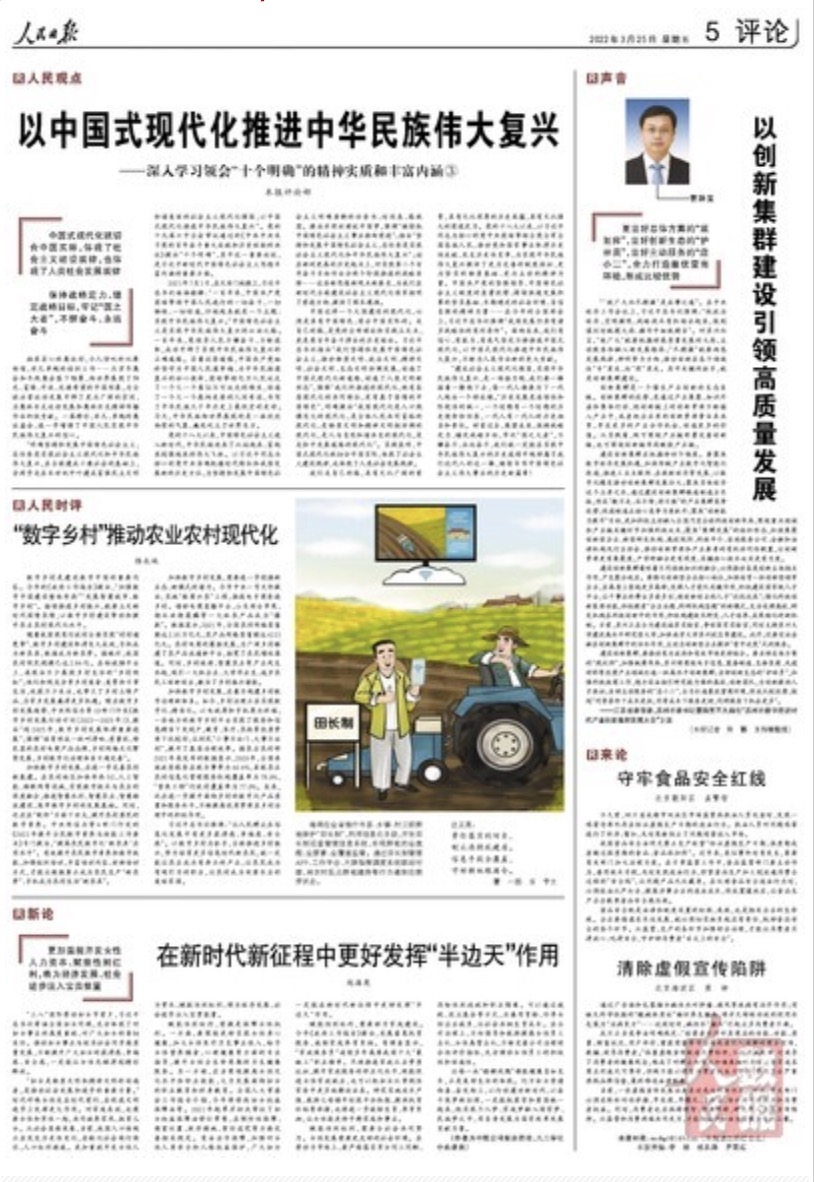 |
